# 

**Published:** 1890-12

**Authors:** 


### CONTENTS FOR DECEMBER, 1890.

ORIGINAL COMMUNICATIONS.

A Few Observations on the Therapeutic Value of Electricity. By ERNEST WENDE, M. D., B. Sc. ... 257
Mechanism of Anemic Murmurs. By DELANCEY ROCHESTER, M. D. ... 262
Note of Subjective Conjunctivitis. By JACOB GOLDBERG, M. D. ... 267

SOCIETY PROCEEDINGS.

Philadelphia County Medical Society ... 268
Buffalo Obstetrical Society ... 274
New York Academy of Medicine ... 279
Calcareous Degeneration of the Hypophysis or Pituitary Body ... 286

TRANSLATIONS.

Cure of Epithelioma of Sebaceous Origin by Resorcin ... 288

PROGRESS IN MEDICAL SCIENCE.

Surgery. By JOHN PARMENTER, M. D. ... 291
Ophthalmology. By ALVIN A. HUBBELL, M. D. ... 293
Some Obstetric Points. By GEORGE H. ROHÉ, M. D. ... 298

CORRESPONDENCE.

Dr. Henry O. Marcy ... 295

EDITORIAL.

Koch's Discovery ... 299
The Southern Surgical and Gynecological Society ... 300
The New York Academy of Medicine ... 301

PERSONAL.

Dr. I. S. Stone—Dr. Seneca D. Powell ... 302

OBITUARY.

Dr. Richard J. Levis—Dr. Arthur B. Carpenter ... 303

SOCIETY MEETINGS.

Buffalo Obstetrical Society ... 303

JOURNALISTIC NOTES.

*Review of Insanity and Nervous Diseases — Annals of Surgery* ... 303

BOOK REVIEWS.

The Essentials of Medical Chemistry ond Urinalysis ... 304
Familiar Forms of Nervous Disease ... 306
The Medical Students' Manual of Chemistry ... 307
The Science and Art of Obstetrics ... 309
Saunders' Question Compends ... 310
Transactions of the American Surgical Association ... 311
Lectures on Massage and Electricity in the Treatment of Disease ... 312
The Medical News Visiting List — Transactions of the Southern Surgical and Gynecological Association ... 313
A Compend of Surgery — A Practical Treatise on Impotence ... 314
The Biography of Ephraim McDowell, M. D. ... 315
Books and Pamphlets Received ... 315
Miscellany ... 319

